# Local Lung Immune Response to *Mycobacterium bovis* Challenge after BCG and *M. bovis* Heat-Inactivated Vaccination in European Badger (*Meles meles*)

**DOI:** 10.3390/pathogens9060456

**Published:** 2020-06-09

**Authors:** Cristina Blanco Vázquez, Miguel Prieto, Marta Barral, Ramón Antonio Juste, Sandrine Lesellier, Francisco Javier Salguero, Dipesh Davé, Ileana Zorhaya Martínez, María Gracia de Garnica García, Rosa Casais, Ana Balseiro

**Affiliations:** 1 Centro de Biotecnología Animal, Servicio Regional de Investigación y Desarrollo Agroalimentario (SERIDA), 33394 Gijón, Asturias, Spain; jmprieto@serida.org (M.P.); rosacg@serida.org (R.C.); 2 Animal Health Department, NEIKER-Instituto Vasco de Investigación y Desarrollo Agrario, 48160 Derio, Bizkaia, Spain; mbarral@neiker.eus (M.B.); rjuste@neiker.eus (R.A.J.); 3 Nancy Laboratory for Rabies and Wildlife (ANSES), 54220 Malzéville, France; sandrine.lesellier@anses.fr; 4 Public Health England, PHE Porton Down, Salisbury SP4 0JG, UK; Javier.Salguero@phe.gov.uk; 5 Bacteriology Department, Animal and Plant Health Agency (APHA), Weybridge KT15 3NB, UK; Dipesh.Dave@apha.gov.uk; 6 Departamento de Sanidad Animal, Facultad de Veterinaria, Universidad de León, 24071 León, Spain; ileanazorhaya.martinez@upaep.mx (I.Z.M.); abalm@unileon.es (A.B.); 7 Facultad de Veterinaria, Universidad Popular Autónoma del Estado de Puebla, UPAEP Universidad, 72410 Puebla, Mexico; 8 Micros Veterinaria, 24071 León, Spain; graciadegarnica@gmail.com; 9 Departamento de Sanidad Animal, Instituto de Ganadería de Montaña (CSIC-Universidad de León), Finca Marzanas, Grulleros, 24346 León, Spain

**Keywords:** tuberculosis, badger, BCG vaccine, *Mycobacterium bovis* heat-inactivated (HIMB) vaccine, immunohistochemistry, granuloma

## Abstract

Tuberculosis (TB) vaccination could be used as a key part of integrated strategies for the disease’s control if an effective and safe vaccine under field conditions is obtained. Recent studies in Spain have evaluated the protective efficacy of two oral vaccines against experimental challenge with live intra-bronchial *Mycobacterium bovis* in captive badgers: the live-attenuated *M. bovis* BCG vaccine (Danish strain) and a heat-inactivated *M. bovis* (HIMB) vaccine. With the objective of increasing the knowledge of the cellular development progress of infection and generating further tools to discriminate between mild and severe TB lesions between and within animals, the immunopathology of tuberculous lesions was studied to characterize the local immune response (cell type profile) within lung granulomas from control (non-vaccinated), BCG vaccinated and HIMB-vaccinated experimentally infected badgers with *M. bovis*. Four immunohistochemical protocols, for the specific detection of macrophages, T lymphocytes, B lymphocytes and plasma cells within TB granulomas in formalin fixed sections of the right middle lung lobe (lobe targeted for the *M. bovis* delivery), were performed. Immunolabelled sections were scanned and five randomly selected areas were analyzed with digital image analysis software. The results were expressed as the proportion of the positively immunolabelled area within the total area of the selected site. Data was analyzed using the statistical analysis software (SAS). In the three treatment groups, macrophages were the most abundant inflammatory cells within the granulomas, followed by B lymphocytes and plasma cells. T lymphocyes were absent in those granulomas. This would suggest a predominance of a non-specific innate response mediated by phagocytic cells over an adaptative humoral immune response. The proportion of macrophages and plasma cells was higher in BCG and HIMB-vaccinated badgers, respectively, suggesting the establishment of an adaptative humoral response in HIMB-vaccinated badgers. The lower bacterial load at the lung level, as well as the volume of lesions in lungs using magnetic resonance imaging in badgers with the HIMB vaccine in relation with local immune response presented, must be highlighted, since it would be an advantage in favor of its use under field conditions in terms of reducing TB transmission and environmental contamination.

## 1. Introduction

Animal tuberculosis (TB) remains as one of the most prevalent infectious diseases causing important economical losses worldwide. It poses a risk to human beings since it can also induce zoonotic TB. It is caused by infection with members of the *Mycobacterium tuberculosis* complex (MTBC), mainly *M*. *bovis* and to a lesser extent *M. caprae*. *Mycobacterium bovis* infects a wide range of domestic animals including cattle (*Bos taurus*) [1], goat (*Capra hircus*) [2] and sheep (*Ovis aries*) [3]. It also infects different species of wild animals, such as brushtail possums (*Trichosurus vulpecula*) [4], African buffalo (*Syncerus caffer*) [5], wild boar (*Sus scrofa*) [6], fallow deer (*Dama dama*) [7] and European badger (*Meles meles*) [8]. In spite of major efforts invested in the control of the disease in cattle, considered the main domestic reservoir, TB is still present in many European countries [9]. In addition, the existence of wild reservoirs makes eradication in livestock more complicated. In Europe, particularly in the United Kingdom (UK) and the Republic of Ireland (ROI), badgers are recognized as major TB reservoir hosts with the potential to transmit infection to cattle herds [10,11], while in Spain the wildlife species most commonly associated with outbreaks in domestic animals is the wild boar [12]. As part of integrated control strategies for the disease, some countries, such as the UK and ROI, have proposed the vaccination of badgers with live-attenuated *M. bovis* bacillus Calmette–Guérin (BCG) vaccine [13,14,15]. A live vaccine (BCG) for badgers is only licensed in the UK for intramuscular delivery, and its use in oral baits is promising, although the use of a live vaccine at a sufficient protective dose complicates the delivery in the field, especially at high environmental temperatures, impacting the survival of the vaccine [16]. The efficacy of a heat-inactivated *M. bovis* (HIMB) vaccine has also been assessed in different species, such as wild pigs [17], red deer [18], wild boar [19,20,21], goats [22,23], sheep [24] and zebrafish [25]. A recent experimental study in Spain showed a similar reduction in bacterial loads and lesion scores in BCG and HIMB orally vaccinated badgers [26] compared with non-vaccinated controls. The use of the HIMB vaccine in field conditions is therefore envisaged, with the benefit of being more stable in baits, and potentially less immunogenic by the oral route for cattle [27], than BCG.

In order to understand the pathogenesis of animal TB, different studies have focused on the study of the development of granulomas and the cell phenotype in each granuloma stage, both in natural and experimental infections. Cell characterization of TB granulomas using immunohistochemistry (IHC) has been performed in species such as wild boar [6], fallow deer [7], pacu [28], sheep [29], cattle [30,31] and badgers [32]. However, the cellular characterization of granulomas in vaccinated animals is poorly understood. Differences in cellular patterns within TB granulomas imply particularities in the host immune response against the pathogen. In this sense, the cell characterization of TB granulomas in control and vaccinated animals might help to determine mechanisms of protection. Previous studies have evaluated differences in cell granuloma composition in BCG vaccinated versus (vs) non-vaccinated cattle [33,34], where BCG vaccination was protective in the sense that it reduced the number and severity of lesions and the bacterial load in tissues. Overall, the number of inflammatory cells was reduced (5–10% for T lymphocytes) in vaccinated animals, and the efficiency in the expression of cytokines with a bactericidal effect was increased [34].

The aims of the present study included (i) the immunohistochemical cell characterization of tuberculous lesions in control (non-vaccinated), BCG and HIMB vaccinated European badgers experimentally infected with *M. bovis*, and (ii) the study of differences in the local cell immune response as a contribution to the understanding of vaccine protection mechanisms.

## 2. Results

### 2.1. Proportion of Total Cells in Treatment Groups

**Figure 1:**
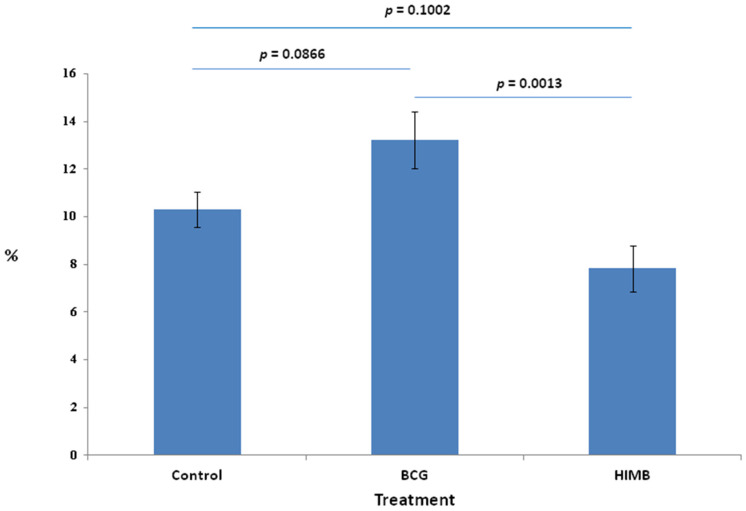
Total cell proportion represented by percentage of immunohistochemical stained area in the control, Bacillus Calmette–Guérin (BCG) and heat-inactivated *Mycobacterium bovis* (HIMB) vaccinated badgers. Treatment mean proportion.

Total cell proportions were significantly different between treatment groups (Figure 1). There were significant differences in the total number of cells (control, 10.30%; BCG, 13.22%; HIMB, 7.82%) between the BCG and HIMB groups (*p* = 0.0013), and almost significant differences between the control and BCG groups (*p* = 0.0866) and the HIMB (*p* = 0.1002) group.

### 2.2. Cell Types and Differences within Treatment Groups

**Figure 2:**
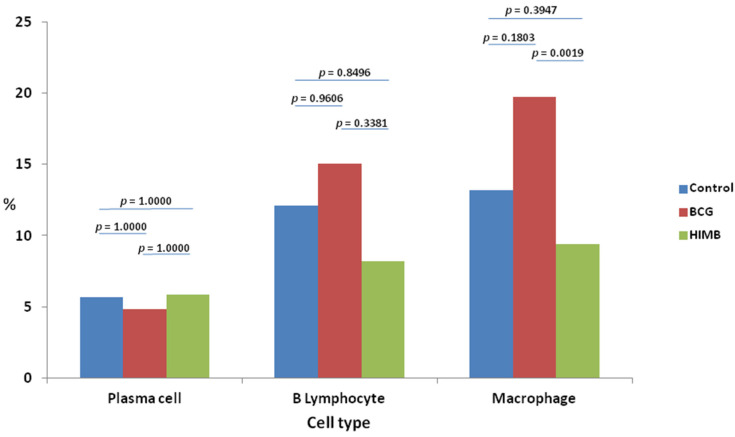
Cell proportion within control, Bacillus Calmette–Guérin (BCG) and heat-inactivated *Mycobacterium bovis* (HIMB) vaccinated animals. Mean cell type proportion within each treatment group.

Macrophages were the most abundant cell type in the three treatment groups (Table 1). Significant differences in macrophages were found between BCG vaccinated and control animals (*p* = 0.044). In addition, there were also significant differences in macrophage proportions between BCG and HIMB vaccinated animals (*p* = 0.0017). The next most abundant cell type was B lymphocytes, followed by plasma cells and T lymphocytes (Figure 2), which were absent in all animals. No significant differences were found between other cell types in any of the groups.

### 2.3. Distribution of Cells according to Granuloma Type (1 to 4)

**Figure 3:**
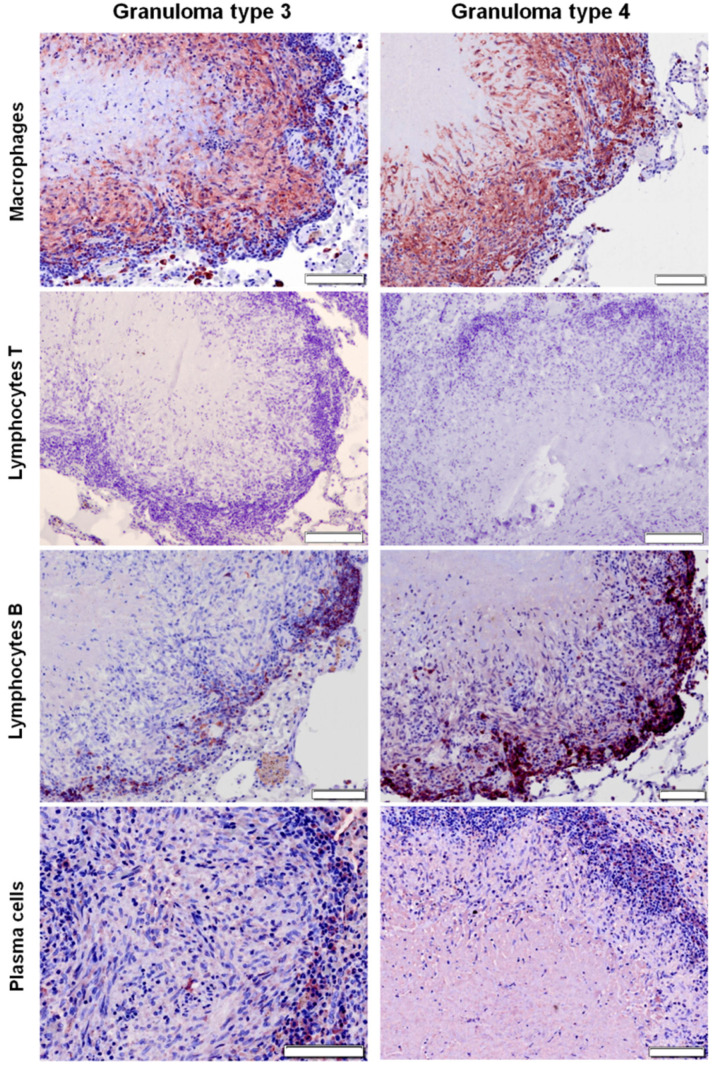
Right middle lung lobe; immunohistochemical characterization (ABC standard) of cellular populations in tuberculous granulomas (types 3 and 4) in control group. Note that macrophages are the most abundant cells followed by B lymphocytes, which are forming clusters in the periphery of the granulomas. T lymphocytes are absent and plasma cells appear sparsely distributed between B cells and macrophages surrounding the necrotic areas. Bar = 100 microns.

**Figure 4:**
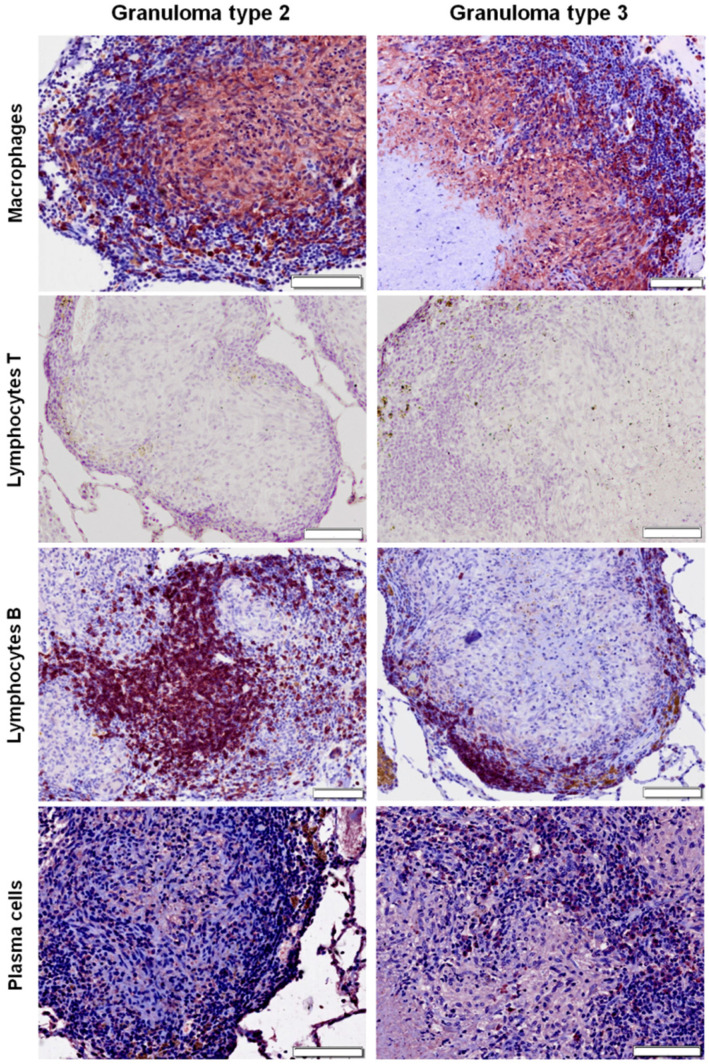
Right middle lung lobe; immunohistochemical characterization (ABC standard) of cellular populations in tuberculous granulomas of bacillus Calmette–Guérin (BCG) vaccinated group (types 2 and 3). Note that macrophages are the most abundant cells followed by B lymphocytes, which are forming clusters in the periphery of the granuloma type 3. In granuloma type 2, cells are diffusely distributed throughout the lesion. Scattered plasma cells are observed within the granuloma types 2 and 3. T lymphocytes are absent. Brown color observed in images from T lymphocytes corresponds to pigment. Bar = 100 microns.

**Figure 5:**
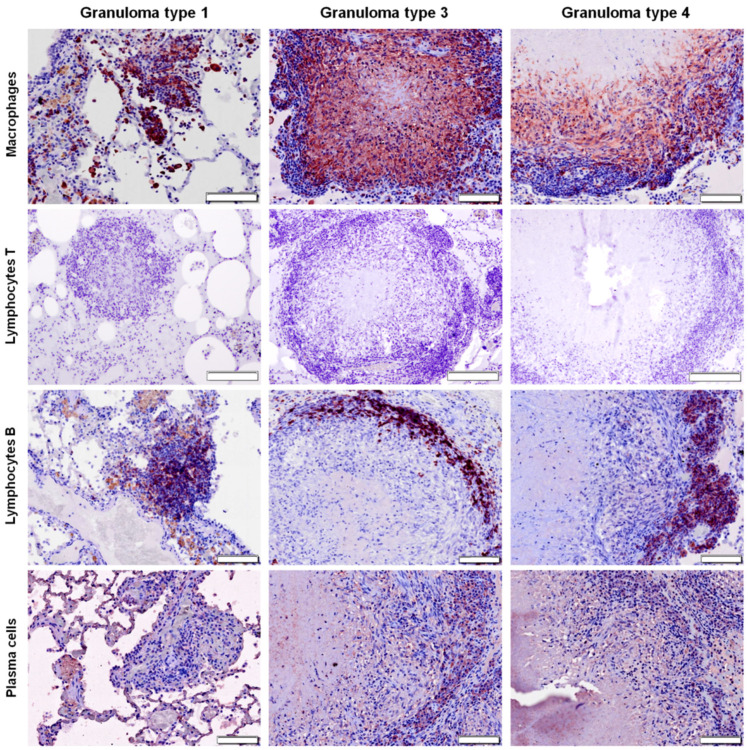
Right middle lung lobe; immunohistochemical characterization (ABC standard) of cellular populations in tuberculous granulomas of heat-inactivated *Mycobacterium bovis* (HIMB) vaccinated group. Note that macrophages are the most abundant cells, followed by B lymphocytes, which are forming clusters in the periphery of granuloma types 3 and 4. Plasma cells appear sparsely distributed between B cells and macrophages surrounding the necrotic areas. In granuloma type 1, cells are diffusely distributed throughout the lesion. T lymphocytes are absent within all types of granuloma. Bar = 100 microns.

In type 1 and type 2 granulomas (see Materials and Methods sections for details), macrophages were diffusely distributed throughout the lesion (Figure 3 and Figure 4), while they were present in the outer layers and surrounding the necrotic foci in more advanced stages (granuloma types 3 and 4) (Figure 3, Figure 4 and Figure 5). B lymphocytes were scattered within the lesion in early stages (granuloma types 1 and 2) (Figure 4 and Figure 5), and forming clusters and surrounding the necrotic area in advanced stages (granuloma types 3 and 4) (Figure 3, Figure 4 and Figure 5). IHC analysis showed very few scattered positively stained plasma cells within the granuloma types 1 and 2 (Figure 4 and Figure 5), but their number increased as the granuloma developed (types 3 and 4). They were found forming clusters or sparsely distributed between B cells and macrophages surrounding the necrotic area (Figure 3, Figure 4 and Figure 5).

### 2.4. Statistical Analysis in Relation with Local Response in Lung

**Figure 6:**
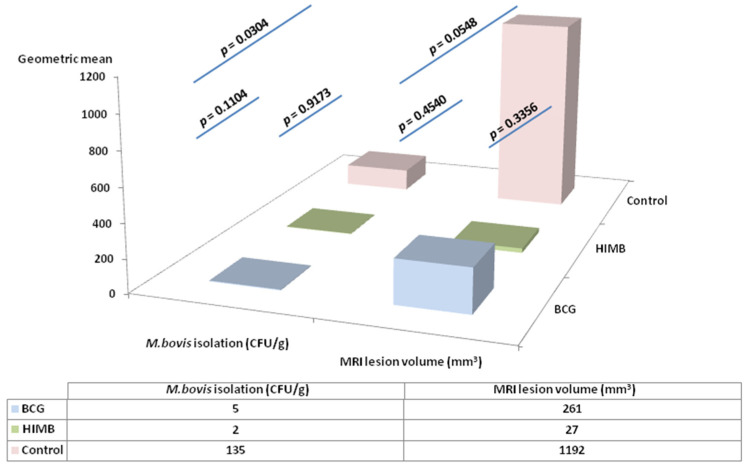
Comparison of bacterial load in mediastine and tuberculosis lesion volume (mm^3^) in lungs between the three treatment groups. BCG: Bacillus Calmette–Guérin; HIMB: heat-inactivated *Mycobacterium bovis* vaccinated animals; CFU: colony forming units; MRI: magnetic resonance imaging.

The results of the isolation of *M. bovis* in mediastine showed significant differences between the three treatment groups (Figure 6). The bacterial load was not different between vaccinated groups (*p* = 0.9173), but was significantly reduced (98.36%; *p* = 0.0304) in the HIMB, and nearly so in the BCG (96.63%; *p* = 0.1104) groups compared to the control group.

Regarding MRI results, HIMB (42.9%) vaccinated animals tended to have smaller TB lesion areas than BCG (60%) and control (83.3%) groups. This corresponded with a reduction in the lesion volume geometric mean in the vaccinated groups compared to the control group; 78.1% for BCG (*p* = 0.454) and 97.7% for HIMB (*p* = 0.0548).

## 3. Discussion

In this study we have examined, for the first time, the local immune response in control (non-vaccinated), BCG and HIMB-vaccinated badgers experimentally infected with *M. bovis*, through cell characterization and quantification within TB lung granulomas. Regarding the number of total cells, different studies have shown that vaccination of animals is associated with a reduction in the local number and severity of lesions, as well as in the amount of inflammatory cells [33,34]. However, other studies showed that protection was related to an increase in the number of cells, i.e., T lymphocytes and macrophages [35,36,37]. In our study, both vaccinated groups with variable numbers of total cells were protected based on visible lesions. 

The cell distribution pattern was similar in all treatment groups. Granulomas showed a high number of macrophages, followed by B lymphocytes and plasma cells. The composition and distribution of cells observed in this study were similar to those already described in badgers [32], except for T lymphocytes, which were not detected in any animal from our study. The absence of T lymphocytes would indicate the predominance of the humoral immune response at the time of slaughter, which could be interpreted as a rapid progression of the infection towards a more specific immune response. Since this occurred in all groups, it can be postulated that the *M. bovis* strain used in the challenge may have also influenced the granuloma cell composition.

T lymphocytes and their secreted cytokines activate macrophages during the cell-mediated immune response [38,39]. In the present study, no T lymphocytes were detected, therefore one possible hypothesis for this result is that the macrophages in granulomas would be those carrying out the non-specific innate response, which would be represented by phagocytic cells rather than by specific adaptative cells [29,37]. However, the lack of histological markers of adaptative immunity, including T cells, is surprising, 12 weeks post-challenge, when peripheral T cell activity was observed [26]. The higher proportion of macrophages in the BCG group may reflect (i) the greater immunogenic properties of the BCG (live vaccine) than HIMB vaccine (inactivated vaccine), or (ii) the stronger stimulation of the innate immune response of the host. It also may, on the contrary, indicate (iii) a failure of the macrophages to efficiently destroy the mycobacteria thriving within the lesion and thus inducing a further influx of inflammatory cells. The latter hypothesis might be supported by the observation that BCG induces TH1/TH17 responses against TB and other unrelated pathogens [40,41,42]. In addition, live vaccines are more immunogenic than inactivated ones [43]. It should be noted the differences between our results and those found in previous studies, in which the number of macrophages decreased in BCG vaccinated animals compared to control ones, which would be consistent with a cellular immune response against *M. bovis* in early stages mediated by T lymphocytes and macrophages [34]. This difference could be due in part to the type of tissue where cellular characterization was performed in each study: lymphoid tissue vs lung tissue. According to this view, the HIMB vaccine could have induced a more effective response in the lungs, which would not have stuck the macrophagic cells in the granuloma, and led to infection clearance. This could be supported by the finding that, in this study, the control badgers presented a high proportion of macrophages within granulomas in advanced stages (types 3 and 4), as seen in previous studies in badgers [32], sheep and pacu fish [28,29], but not in fallow deer, wild boar or cattle at advanced stages of the disease [6,7,30]. A detailed comparison of the role of macrophages across host species may highlight the importance of this cell type in TB inflammatory responses and the overall severity of the disease.

T lymphocytes, however, were absent in all treatment groups. T lymphocytes have constantly been described as a very abundant cell type in the TB granulomas in badgers [32,44], and also in other species, such as cattle [31,34] or fallow deer (7). In general, in badger studies, the interferon-gamma (IFN-γ) production and peripheral blood mononuclear cells (PBMC) proliferation, measured by lymphocyte transformation assays (LTA), decrease between 12 and 21 weeks post-infection [45,46]. As described in our previous study, IFN-γ producing cells responded to combined PPD-B (bovine purified protein derivate) and PPD-A (avian purified protein derivate) antigenic stimulation by whole blood IFN-γ release assay (IGRA) enzyme-linked immunosorbent assay (ELISA) and IGRA enzyme-linked immunospot assay (ELISPOT), 2 weeks post-infection, peaking at 7 weeks post-infection and then decreasing. Necropsies were performed at week 12 post-infection, which might explain the absence of T lymphocytes. In this case, our results are similar to those found in a previous study [34], since a decrease of T lymphocytes was observed after BCG vaccination, consistent with a cellular immune response against *M. bovis* in the early stages mediated by T lymphocytes and macrophages. If we compare our control animals with the cell types found in other animals in advanced granulomas (types 3 or 4), our results are similar to cattle and sheep, where the number of T lymphocytes decreased as the lesion progressed [29,34]. Nevertheless, we have observed great differences with fallow deer, wild boar, and with previous studies of badgers, where the amount of T lymphocytes was very high in advanced stages [6,7,32].

The number of B lymphocytes observed was higher in the BCG group and lower in the HIMB-vaccinated animals, compared to the control group. B cells are involved in the host’s early interactions with intracellular bacterial pathogens, and also participate in the induction of innate defense responses [47]. We found more B lymphocytes in BCG vaccinated badgers than in control animals, similar to what was observed in previous studies in cattle [34]. The increased expression of B lymphocytes within the granulomas, coupled with the high IFN-γ production throughout the course of the experiment, implies that a Th0 response developed in the BCG animals following infection [34]. Higher numbers of B lymphocytes in advanced stages in control animals have also been observed in cattle, sheep and fallow deer [7,29,34]; however, they were fewer in previous studies of badger and wild boar, where the number of B lymphocytes decreases as the granuloma progresses [6,32].

Interestingly, the number of plasma cells was higher in the HIMB group than in the BCG and control groups. Overall, HIMB vaccinated animals presented fewer macrophages and B lymphocytes than BCG vaccinated animals, but a greater number of plasma cells, which suggests that the HIMB vaccine induces the establishment of an adaptative humoral-type response. This fact is in agreement with a previous study in wild boar, which concluded that oral vaccination with HIMB induced both an activation of the innate immune response and an adaptative antibody response [48]. As the infection progresses and the mycobacteria persist in the lesions, the antibody responses also persist or increase [49]; thus the number of plasma cells is expected to be higher in more advanced stages than in early ones. The humoral immune response in badgers is related to the severity of lesions being more evident in advanced stages of the disease [50]. Usually, in experimentally infected badgers, the serological responses develop gradually from 4 to 6 weeks post-infection [26,51], and persist at the time of slaughter 12 weeks post-infection. This fact agrees with previous studies on IHC characterization in TB granulomas in badgers, which suggest that humoral immunity is not a major factor in the early establishment of lesions [32]. Also, the amount of plasma cells found in control badgers is similar to those observed in other species, such as fallow deer and sheep, increasing their numbers as the granuloma progresses [7,29].

It is important to highlight the significant reduction of the bacterial load in the mediastine of the vaccinated animals, with respect to the control group, mainly in the HIMB group. This could be related to the cell ratio findings discussed above, and in any case supports the idea that the HIMB vaccine could have induced a trained immune response [52] that could have better cleared the initial infection. More striking, though, is the fact that for global isolation including all tissues, the bacterial load was higher for the HIMB group than for the BCG group [26]. This shows that *M. bovis* induces a complex immune response highly dependent on the individual host response in each organic region. The HIMB vaccine appears to induce a greater response at the lung level compared to the lymphatic system, which could be crucial for the reduction of TB lesions, as demonstrated both by magnetic resonance image and bacterial load in this particular tissue [26], and thus for a reduction of the excretion of mycobacteria via secretions and aerosol. On the other hand, this fact also justifies in vivo experimentation, and highlights the importance of these experimental infections for understanding this complex system, where different criteria may be needed for the local or general evaluation. These differences, between lung tissue and lymphoid tissue bacterial load, as related to vaccine protection, are probably crucial and require further research.

In conclusion, this study has characterized the immunopathology of the TB granulomas in control, BCG and HIMB vaccinated European badgers, experimentally infected with *M. bovis* in the lung, highlighting the higher proportion of macrophages than B lymphocytes and plasma cells, and the absence of T lymphocytes, which would suggest the predominance of a non-specific innate response mediated by phagocytic cells over an adaptative humoral immune response. In this sense, the HIMB vaccine induced a greater presence of plasma cells than the BCG vaccine did, suggesting the establishment of an adaptative humoral response; although more studies would be necessary to confirm this hypothesis. However, the decrease in the bacterial load and volume of lesions at the lung level with the HIMB vaccine must be highlighted, since it disagrees with results regarding lymphoid tissue bacterial load and lesions [26], which would be an advantage in favor of its use in field conditions for reducing TB transmission and environmental contamination.

## 4. Materials and Methods 

### 4.1. Ethical Statement

All methods were employed in accordance with the relevant guidelines and regulations. All experimental protocols were approved by the ethical committees from Servicio Regional de Investigación y Desarrollo Agroalimentario del Principado de Asturias (SERIDA) and from the Government of the Principality of Asturias. The license reference numbers were 010/07-01-2011, PROAE 20/2015 and PROAE 47/2018.

### 4.2. Animals and Tissue Samples

Samples collected in a previous experimental study were used [26]. Briefly, 24 badgers (13 males and 11 females) were trapped and randomly allocated to three treatment groups: unvaccinated controls (n = 12), oral vaccinated with live Danish BCG [10^8^ colony-forming units (CFU), n = 5], and oral vaccinated with HIMB (10^7^ CFU, n = 7). After a period of 13 weeks post-vaccination, all badgers were experimentally challenged by the endobronchial route in the right middle lung lobe with 1 mL of a *M. bovis* field strain (SB0339) [10^3^ CFU/mL in phosphate buffer saline (PBS)] isolated from a tuberculous wild boar. After a period of 12 weeks post-challenge, badgers where euthanized and a complete collection of tissue (lymph nodes and organs) samples [26] was performed at post mortem examination (PM). In that study, 4 animals (1 control, 1 BCG vaccinated and 2 HIMB vaccinated) were positive by the P22 ELISA on the day of challenge, which would indicate a previous sensitization to *M. bovis* or other mycobacteria not detected using cellular immune techniques [26]. Those animals have not been included in the present study.

### 4.3. Histopathological, Bacteriological and Magnetic Resonance Imaging (MRI) Data

As already reported in a previous publication, samples were processed by standard protocols for histopathology [hematoxylin and eosin (HE) and Ziehl–Neelsen (ZN) staining, specifically for acid-fast bacilli (AFB)], bacteriology and MRI [26]. Only local results from lungs and mediastine have been used for the specific current statistical analyses.

The histological score was calculated for each tissue and the granulomas were classified based on cell composition, severity and number of mycobacteria from types 1 to 4. Briefly, type 1 granuloma consisted of clusters of lymphocytes, epithelioid cells and plasma cells. Granulomas type 2 and 3 presented the same cellular types as granuloma 1, but they also showed coagulative and caseous central necrosis, respectively. Finally, granuloma type 4, the most severe, showed caseous and mineralized central necrosis [26]. The histological score was based on the most severe lesion observed on the section (see [26] for details). The formalin fixed lungs were scanned by MRI in order to determine the lesion volume in lung (mm^3^) in each animal [26]. In Table 2, results obtained in the previous study regarding histological scores, bacterial load and MRI (mm^3^) of each badger are shown. 

### 4.4. Immunohistochemistry (IHC)

The numbers of macrophages, T lymphocytes, B lymphocytes and plasma cells within the TB granulomas located in the right middle lung lobe were calculated. We selected the right middle lung lobe because that tissue was consistently affected (as it was the challenge tissue target) and had a similar size in all studied badgers. The specific details of the IHC protocols are summarized in Table 3. Formalin fixed right middle lung lobe samples were cut into 3-µm sections, dewaxed, rehydrated and rinsed with tap water at room temperature. Afterwards, epitope demasking was performed microwaving in citric acid buffer [1.48 g citric acid (Merck KGaA, Darmstadt, Germany) in 500 mL distilled water] (Table 3). The sections were then washed with tap water or tris buffered saline 1× [TBS 1×, 5 mM Tris (Merck KGaA, Darmstadt, Germany)/HCl (Panreac Química, SLU, Barcelona, Spain) pH 7.6, 136 mM NaCl (Merck KGaA, Darstadt, Germany)] in the case of Lambda light chain primary antibody. Then, slides were placed in different solutions to block endogenous peroxidase activity (Table 3) and washed with tap water or TBS 1× in the case of Lambda primary antibody. Afterwards, the samples were treated to prevent primary antibody cross-reactivity with tissue constituents over a 20-min incubation period at room temperature, with 10% normal horse serum for CD3 protocol (Vector Laboratories, Burlingame, CA, USA) or with 10% normal goat serum for CD20, IBA-1 and Lambda light chain protocols (Vector Laboratories), and 3% bovine serum albumin (BSA, Sigma-Aldrich, Saint Louis, MO, USA). Then, sections were incubated overnight at 4 °C with commercial monoclonal and polyclonal antibodies (Table 3), and then washed three times with TBS 1×. Afterwards, samples were incubated with the appropriate biotinylated secondary antibody (anti-mouse biotinylated in horse or anti-rabbit biotinylated in goat; Vector Laboratories) before being washed three times with TBS 1×. The sections were incubated for 30 min (and 40 min in the case of Lambda primary antibody) at room temperature with Avidin Biotin Complex (ABC kit Peroxidase Standard, Vector Laboratories). Finally, the sections were washed three times with TBS 1× and the signal was detected using Vector® NovaRED™ peroxidase substrate kit SK-4800 (Vector Laboratories) for 2–5 min. Samples were rinsed with tap water or TBS 1× for 5 min, before being placed in Mayer’s hematoxylin (MerckKGaA, Darmstadt, Germany) counterstain for 30 seconds. Finally, the slices were washed with tap water or TBS 1× and mounted with DPX (MerckKGaA, Darmstadt, Germany). Appropriate controls were included in each IHC run. Those included positive controls (lymph nodes from badger where the target antigen was present in the section and the specific antibody was used) (Supplementary Figure S1) and negative controls (slides with omission of the primary antibody). 

### 4.5. Image Analysis 

Immunolabelled sections were scanned at 40× using an Olympus BX51 microscope (Olympus, Tokyo, Japan) with an Olympus XC10 camera (Olympus, Tokyo, Japan). Scanned images were analyzed with digital image analysis software (Nikon NIS-Elements Br, Nikon, Japan). For each animal, five randomly selected areas were sourced as regions of interest (RI) and were analyzed using 200× magnification. The area with an immunohistochemical-positive reaction within RI was calculated by the Nikon NIS-Elements software after setting the thresholds. The results were expressed as the proportion of the positively immunolabelled area within the total area of the selected site.

### 4.6. Statistical Analyses

Data was analyzed using the SAS statistical package (SAS Inc., Cary, NC, USA). To normalize the data for statistical analysis, an arc sin square root transformation was applied to all the proportions. Results were submitted to analysis of variance with the geometrical linear mean (GLM) procedure to determine the statistical significance of differences in the cell type area proportions among treatment groups, and also to determine if there were differences among cell type proportions within each treatment group. Therefore, the resulting model had as independent variables (main effects) the vaccine treatment, the cell type and its interaction, and as dependent variable the stained area’s proportion. A correlation analysis was also run between cell type proportions in order to identify associations between them. The multiple comparison adjusted Tukey–Kramer test was applied for effect level comparison. Statistical significance was accepted at *p* > 0.05.

## Figures and Tables

**Table 1:** Proportion (%) of cellular types found in tuberculous granulomas.

Treatment Group	Badger ID	Macrophages (%)	T Lymphocytes (%)	B Lymphocytes (%)	Plasma Cells (%)
Control	0369	21.86	0	11.242	9.612
Control	0866	7.794	0	7.524	6.72
Control	1464	25.628	0	6.82	3.252
Control	1661	3.406	0	13.48	2.678
Control	2191	6.59	0	27.3	2.14
Control	3959	12.146	0	2.736	5.366
Control	5225	8.606	0	12.962	5.852
Control	5360	28.176	0	15.856	6.852
Control	5338	13.286	0	15.018	5.978
Control	8248	7.662	0	14.362	8.146
Control	9761	8.26	0	8.146	6.382
BCG	2966	14.926	0	29.774	4.1125
BCG	5570	12.098	0	13.8	5.09
BCG	5179	25.46	0	7.966	6.17
BCG	9538	26.52	0	8.726	3.22
HIMB	3902	3.524	0	5.61	2.122
HIMB	4327	8.102	0	7.426	7.96
HIMB	7228	5.172	0	8.084	5.814
HIMB	7603	21.208	0	15.088	11.542
HIMB	9413	9.042	0	7.48	4.144

BCG: Bacillus Calmette–Guérin vaccinated animals; HIMB: heat-inactivated *Mycobacterium bovis* vaccinated animals; ID: identification.

**Table 2:** Histological score, bacterial load and volume of lesion (mm^3^) in lungs using magnetic resonance imaging (MRI) of each badger used (*n* = 20) in the present study (see [26] for details).

Treatment	ID	Right Middle Lung Lobe Histology Score	Lung (Mediastine) *M. bovis* Isolation		Lung MRI (mm^3^)
			COLGRAM	SCO10	
Control	0369	4	50.51	3.92	638
Control	1464	4	5314.53	8.58	573
Control	2191	3	0.00	0.00	464
Control	5338	4	1860.96	7.53	3388
Control	5360	4	0.00	0.00	836
Control	0866	4	48.03	3.87	9653
Control	1661	4	110.13	4.70	9439
Control	3959	4	3400.81	8.13	16,230
Control	5225	4	746.27	6.62	4885
Control	8248	4	263.95	5.58	1982
Control	9761	3	151.52	5.02	0
BCG	2966	2	0.00	0.00	772
BCG	5179	3	0.00	0.00	1503
BCG	5770	3	428.57	6.06	4007
BCG	9538	2	0.00	0.00	0
HIMB	3902	1	0.00	0.00	0
HIMB	4327	3	0.00	0.00	0
HIMB	7228	4	53.19	3.97	0
HIMB	7603	4	0.00	0.00	3721
HIMB	9413	3	0.00	0.00	4125

BCG: Bacillus Calmette–Guérin vaccinated animals; HIMB: heat-inactivated *Mycobacterium bovis* vaccinated animals; ID: identification; COLGRAM: colony forming units (CFU) of *M. bovis* per gram; SCO10: mean log10 CFU score.

**Table 3:** Immunohistochemical protocols used for cellular type characterization.

Primary Antibody				Epitope Demasking	Block Endogenous Peroxidase Activity	Secondary Antibody		
Description	Specificity	Source	Dilution			Description	Source	Dilution
**IBA-1**	Macrophages	Wako Pure Chemical Industries, Osaka, Japan. Catalog number: 019-19741	1/1200	Microwave in citrate pH 6.0 4 × 5 min	Methanol (VWR, Monroeville, PA, USA) with 3% hydrogen peroxide (Sigma-Aldrich, MO, USA) 10 min room temperature	Anti-rabbit biotinilated in goat	Vector Laboratories, Burlingame, CA, USA	1/200
**CD3**	Pan T cell	Novocastra Leica, Newcastle, UK. Catalog number: NCL-L-CD3-565. Clone: LN10	1/400	Microwave in citrate pH 6.0 5 × 5 min	Methanol (VWR, Monroeville, PA, USA) with 3% hydrogen peroxide (Sigma-Aldrich, MO, USA) 10 min room temperature	Anti-mouse biotinilated in horse	Vector Laboratories, Burlingame, CA, USA	1/200
**CD20**	Pan B cell	ThermoFisher, Waltham, MA, USA. Catalog number: PA5-16701	1/500	Microwave in citrate pH 6.0 4 × 5 min	Methanol (VWR, Monroeville, PA, USA) with 3% hydrogen peroxide (Sigma-Aldrich, MO, USA) 10 min room temperature	Anti-rabbit biotinilated in goat	Vector Laboratories, Burlingame, CA, USA	1/200
**Lambda light chain**	Plasma cells	Novocastra Leica, Newcastle, UK. Catalog number: NCL-L-LAM-578. Clone: SHL53	1/200	Microwave in citrate pH 6.0 4 × 5 min	Destiled water with 0,5% hydrogen peroxide (Sigma-Aldrich, Saint Louis, MO, USA) 30 min room temperature in obscurity	Anti-rabbit biotinilated in goat	Vector Laboratories, Burlingame, CA, USA	1/200

## Supplementary Materials

The following are available online at https://www.mdpi.com/2076-0817/9/6/456/s1, Figure S1: Immunohistochemical technique in positive controls; lymph nodes from badger.
