# Supplementary material for: Local Lung Immune Response to Mycobacterium bovis Challenge after BCG and M. bovis Heat-Inactivated Vaccination in European Badger (Meles meles)
Source: Pathogens. 2020 Jun 9;9(6):456. doi: 10.3390/pathogens9060456 (PMC7350352; doi:10.3390/pathogens9060456)
Supplement: Supplementary file 1 [file pathogens-09-00456-s001.pdf]

Supplementary Materials: The following are available online at www.mdpi.com/xxx/s1.

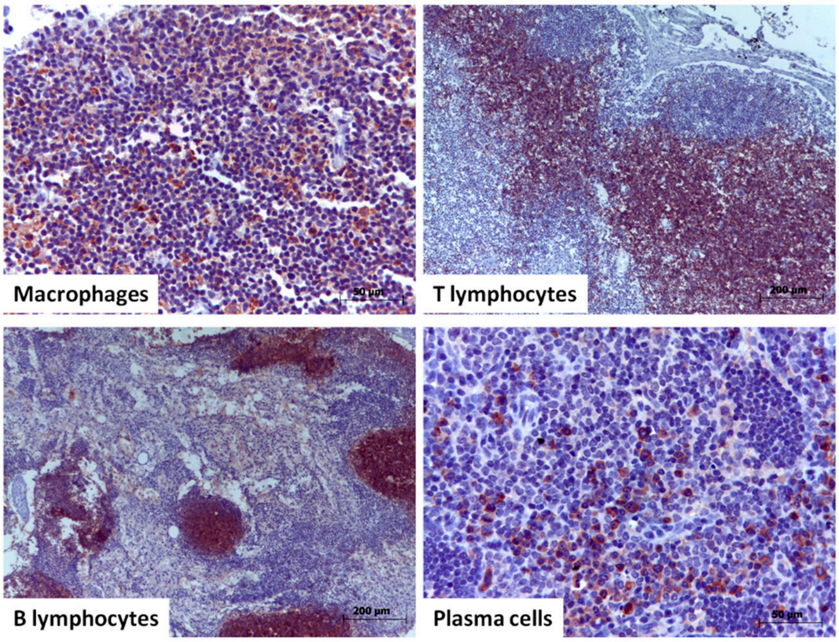

Figure S1. Immunohistochemical technique in positive controls; lymph nodes from badger.
